# Assessment of Environmental Attitudes and Risk Perceptions among University Students in Mersin, Turkey

**DOI:** 10.1155/2017/5650926

**Published:** 2017-08-22

**Authors:** Gulcin Yapici, Oya Ögenler, Ahmet Öner Kurt, Fazıl Koçaş, Tayyar Şaşmaz

**Affiliations:** ^1^Department of Public Health, Faculty of Medicine, Mersin University, Mersin, Turkey; ^2^Department of History of Medicine and Ethics, Faculty of Medicine, Mersin University, Mersin, Turkey

## Abstract

**Background:**

Environmental destruction is one of the most important problems in this century.

**Objective:**

The aim of the study was to determine the environmental attitudes and perceived risks associated with environmental factors of the students.

**Methods:**

This cross-sectional study was conducted in 7 faculties of Mersin University. The research data were collected using a questionnaire. The questionnaire included sociodemographic characteristics, the “Environmental Attitudes Scale,” and the “Environmental Risk Perception Scale.” 774 students who filled out questionnaires were evaluated.

**Results:**

The sample included 55.8% females. Environmental Attitudes Scale mean scores of students were identified as 81.1 ± 11.3. The highest perceived risk was release of radioactive materials associated with nuclear power generation. The environmental attitudes and risk perception scores were higher in Health Sciences than in the other faculties. Females were more positive towards the environment and had higher risk perceptions than the men. There is a negative correlation between age and resource depletion risk and global environmental risk score.

**Conclusion:**

Students had a positive attitude to the environment and had moderate-level risk perception about the environment. Environmental awareness of students, especially those studying in the Social Sciences, should be increased. The environmental education curriculum should be revised throughout all the courses.

## 1. Introduction

In the past 50 years, people have become more aware of the problems originating from the interaction between humans and the environment. In this regard, governments, nongovernmental organizations (NGOs), international institutions, and individuals are encouraged to show enthusiasm towards environmental protection and development [1, 2]. In addition to the regulatory frameworks associated with the environment, individuals also have responsibilities. The behavior of individuals follows a course parallel to the attitude and risk perception towards the environment. Attitude can be defined as “*a settled way of thinking about characters, objects and subjects*” [3]. Attitude is comprised of three components: cognitive (knowledge and beliefs), affective (emotional response), and behavioral (past and present behavioral response). These three components are also associated with risk perception [4]. The term* risk *denotes a possibility of suffering injury or disease due to a certain factor and the severity of this injury or disease [5]. The answer to the question “Why some people perceive some hazards more risky than others?” involves many factors associated with risk perception including age, gender, educational status, income, scientific education, religious beliefs, political views, cultural background, personal experience with hazards, values, social confidence, anxiety, self-reliability, general beliefs, environmental beliefs, and personal viewpoints, all of which may have an influence over risk perception. Individual environmental risk perception and attitude should influence the behavior of solving environmental problems [6–8].

In this regard, the opinions of university students who will face environmental problems at some level and play a role in their solutions in the future are import. There are few studies assessing university students risk perception and attitudes towards the environment in Turkey. Our study was designed to determine the attitudes and risk perceptions and to increase the environmental awareness of students at the Mersin University.

## 2. Materials and Methods

This cross-sectional study was conducted in two campuses of the Mersin University in Turkey. 1971 final-year students were enrolled at 14 faculties and institutions with 4-year or longer programs on campus in the 2014 school year. With a 99% confidence interval (CI), a 3% margin of error, and 50% prevalence, the minimum sample size needed a total of 945 students (Epi Info version 3.5.1). We aimed to reach 950 students. Seven faculties were randomly selected and were split into 4 groups: Faculty of Medicine and Nursing School (Health Sciences), Faculty of Engineering and Faculty of Aquacultural Engineering (Science Engineering and Technology), Faculty of Economic and Administrative Sciences and Faculty of Tourism (Social Sciences), and Faculty of Education (Educational Sciences).

The data were collected between May and June 2014. The researchers met with students in their classrooms. After receiving information about the study, a written informed consent was obtained from participants. Questionnaires were then filled out by the participants. We reached 918 of 950 students whom we planned to access (accession rate, 96.6%). Of them, 113 students refused to participate in the study (rejection rate, 12.3%). The 805 students who participated in the study fulfilled the survey form. 31 forms had to be excluded because participants did not answer all questions. We performed our assessment based on the remaining 774 questionnaires (participation rate, 81.5%).

The study questionnaire comprised three sections. The first section included 22 questions on sociodemographic characteristics (sex, age, mother's and father's education level, mother's and father's profession, family monthly income, monthly allowance of the students, places of residence, and the faculty they attended), environmental education status (received environmental education in the primary/secondary school, university, or extracurricular activity), and interest in environmental subjects. The second section included the 21-question “*Environmental Attitudes Scale*” (EAS), devised by Şama [9]. EAS is a 5-point Likert scale. The maximum score was 105, while the minimum score was 21. The responses to the attitude subset of the questionnaire were scored 5, 4, 3, 2, and 1 corresponding to Strongly Agree, Agree, Undecided, Disagree, and Strongly Disagree, for positive statements (10 items). The values were reversed for negative items (11 items). The third section comprised the 22-question “*Environmental Risk Perception Scale*” (ERPS), adapted to Turkish by Altunoğlu and Atav [10] from the original work of Slimak and Dietz [11]. The ERPS was a 7-point Likert scale (1, not important; 4, moderately important; 7, extremely important). The scale consists of 4 factors which were ecological risks (items (10), (12), (13), (14), (15), (16), (17), and (18)), chemical waste risk (items (5), (6), (7), (8), (9), and (11)), resource depletion (items (19), (20), (21), and (22)), and global environmental risks (items (1), (2), (3), and (4)). The risk items are listed in Table 2 according to number sequence.

The study was approved by the Ethics Committee of Clinical Researches and Administration of Mersin University.

EAS and ERPS were analyzed as the dependent variables, while the sociodemographic characteristics and the data concerning the environmental education status were used as independent variables. The results were expressed as frequency, mean ± standard deviation, and median values. The paired comparisons were performed using Student's *t*-test or Mann-Whitney *U* test, as appropriate, whereas the multiple group comparisons were carried out with the ANOVA test. Two responses were assessed with Pearson's correlation test. *p* < 0.05 was recognized as statistically significant.

## 3. Results

Among the 774 students, 432 (55.8%) were female and 342 (44.2%) were male. The mean age was 23.6 ± 1.8. A total of 380 (49.4%) students were living in a metropolitan city. While 298 (38.9%) students were living together with their friends, 222 (29.0%) were living with their family. Regarding the students' families, 482 (64.3%) of their mothers and 338 (44.9%) of their fathers were primary school graduates or were uneducated. 600 (80.9%) of the mothers were not working. The median annual income of the families was 2000 Turkish Liras (TL) (approx. 646 Euro) and the median monthly allowance of the students was 500 TL (approx. 160 Euro) (Table 1).

Among the students, 225 (29.0%) were in the Social Sciences, 201 (26.0%) were in the Health Sciences, 188 (24.3%) were in the Science Engineering and Technology, and 160 (20.7%) were in the Educational Sciences. A total of 408 (53.0%) students had received environmental education at the university. 623 (80.8%) students were interested in environmental subjects, 111 (14.4%) were members of an environmental organization, and 327 (42.4%) were participating in environmental activities (Table 1). The sources of knowledge for environmental problems were the Internet in 676 (87.6%), TV in 450 (58.3%), newspapers, books, magazines, and articles in 399 (51.7%), and NGOs in 224 (29.0%).

The mean EAS score was 81.1 ± 11.3 (min. 33, max. 105). The distribution of the mean scores was as follows: 83.3 ± 10.9 for Health Sciences, 81.7 ± 10.3 for Life Sciences, 80.9 ± 11.3 for Educational Sciences, and 78.8 ± 12.2 for Social Sciences. The mean EAS score was significantly higher in Health Sciences than in Social Sciences (*p* = 0.001). Also, the mean EAS score in women (83.1 ± 10.1) was significantly higher than that in men (78.6 ± 12.3) (*p* < 0.001). There was a significant correlation between the EAS score and the household profile. The students living with their parents had higher EAS scores than those living with their friends. The EAS score did not show any significant correlation with participant age, educational background of parents, occupation of parents, family monthly income, student monthly allowance, and place of longest residency (*p* > 0.05).

Moreover, the EAS score showed no correlation with taking environmental courses before or during university, membership in an environment-related NGO, and participation in environmental activities. However, the students interested in the environment (82.3 ± 11.0) had significantly higher EAS scores than those who were not interested (*p* < 0.001).

The mean scores of each item in the ERPS are shown in Table 2. Students described radioactive spillage from nuclear energy facilities as the highest risk and commercial fishing as the lowest risk (Table 3).

Subcategory assessment of the ERPS yielded the following results: chemical waste risk (5.8 ± 1.2), global environmental risk (5.6 ± 1.2), ecological risks (5.5 ± 1.1), and risk of resource depletion (4.9 ± 1.3).  Table 4 shows the mean scores relative to the faculties. There were significant differences between the faculties with regard to ecological risks, chemical waste risk, and global environmental risk. The mean score for ecological risk was higher in the Health Sciences than in the Social Sciences; the mean score for chemical waste risk was higher in the Health Sciences than in the Social Sciences and Educational Sciences, while being higher in the Science Engineering and Technology than in the Social Sciences; the mean score for global environmental risk was lower in the Social Sciences than in the Health Sciences, Science Engineering and Technology, and Educational Sciences. There was no difference between the faculties with regard to the risk of resource depletion.

Regarding the relation between age and the risk factors, increasing age showed a negative correlation with risk of resource depletion (*p* = 0.005) and global environmental risk (*p* = 0.03). Women displayed higher mean scores for all risk factors as compared to men (*p* < 0.001). The students with a working mother had higher scores for ecological risk, chemical waste risk, and global environmental risk as compared to those with nonworking mothers (*p* = 0.04, *p* = 0.03, and *p* = 0.05, resp.). The four risk factors exhibited no significant difference with regard to parents' educational statuses, father's occupation, family monthly income, student's monthly allowance, and preuniversity place of residence (*p* > 0.05).

There was no significant difference between the four risk factors with regard to presence of preuniversity education and/or extracurricular courses on environment (*p* > 0.05). The scores were higher in students interested in environmental issues than in students not interested, and the scores were also higher in students taking part in environmental activities than in those not taking part (*p* < 0.05) (Table 5). However, being a member of an environmental NGO was not associated with a difference with regard to risk factor mean scores.

Total EAS mean scores and mean ERPS scores showed a positive correlation. Increasing EAS scores were associated with increasing scores for the four risk factors (ecological risks, *r* = 0.42, *p* < 0.001; chemical waste risk, *r* = 0.51, *p* < 0.001; resource depletion, *r* = 0.23, *p* < 0.001; global environmental risk, *r* = 0.46, *p* < 0.001).

## 4. Discussion

In our study, the mean EAS score was 81.1. Since the maximum score that can be achieved in the scale is 105, our students had a positive attitude towards the environment. However, it could be improved.

According to the students, the highest environmental risk was “radioactive waste.” Our study was conducted in Mersin which is the province where the first nuclear power plant of Turkey is planned to be built. This may be the reason why the students regard radioactive waste as the most hazardous environmental threat. It is considered that the risk perception is higher if the hazard is close to where the students live.

Human behavior towards the environment is shaped by a comprehensive perception about the interaction between man and nature. The interaction between man and nature is evaluated from two viewpoints: human-centered and nature-centered. Human-centered viewpoint regards nature as an immense resource for human use. However, nature-centered viewpoint does not agree with such an approach and takes nature as an entity of its own. The very existence of nature, first and foremost, serves itself [12]. In the current study, the positive attitude of students towards the environment was representative of a human-centered viewpoint. They regarded chemical waste as the top environmental risk that largely affects human life. Ecological and global environmental risk items, mostly reflecting a nature-centered or holistic approach, gained moderate scores, while the items involving the resource depletion had the lowest scores.

In our study, the attitude and risk perception of the students were observed to differ depending on their faculty. Students in health-related faculties had significantly higher EAS scores as compared to those attending faculties of Social Sciences. Students of Health Sciences demonstrated the highest ERPS scores, while students of Social Sciences showed the lowest ERPS scores. Among the students of Health Sciences, the five items with highest ERPS scores directly involve people's health. The fact that students of Health Sciences exhibited more positive attitudes towards the environment, with highest risk perception compared to all other students, may be particularly associated with their education and occupational proficiency. The students of the educational faculty deemed different environmental risks more important including chemical waste, as well as global and ecological risk factors. This may be due to the presence of environmental issues in the curriculum of the Faculty of Educational Sciences. The students in this faculty will be future teachers and will influence the views of future generations. Students of the Social Sciences, deemed as the probable owners of the future decision-making positions, had the lowest scores for environmental attitude and risk perception. As future administrators, the students of Social Sciences should have adequate information about the environmental issues in order to gain the ability to develop solutions.

In our study, the attitude and risk perception of women were higher than those of men. Studies investigating the attitude, behavior, and risk perception of people have yielded varying results on the influence of gender. While some studies found that women had a higher level of risk perception and positive attitude towards the environment as compared to men [13–18], some did not find any difference with regard to gender difference [11, 19]. Women generally have a higher level of anxiety for health and safety, thus feeling more susceptible to environmental problems. This may be associated with more positive attitudes and higher sensitivity towards potential risks.

In our study, we found a correlation between age and environmental risk perception. As mentioned in some studies, more positive environmental attitude and higher risk perception are expected with increasing age [11, 18, 19]; however, our study showed decreasing resource depletion and global environmental risk scores with increasing age. This result shows that environmental negativities of our students have a tendency to increase during their professional lives.

Prior to the study, we had expected that higher parents' educational status and income level would yield higher levels of environmental awareness. However, these parameters were observed to have no effect on the environmental attitudes of the students. Nonetheless, EAS scores of the students living with their parents were higher than those living with their friends. The reason behind this difference may be hearing environmental discussions within the family or easier access to communication tools such as newspapers and television.

In our study, mother's working status was found to have no impact on the environmental attitude; however, those with a working mother had higher ecological and chemical waste risk scores as compared to those with nonworking mothers. Maybe it is because working mothers are more in touch with the environment, which contributes to their environmental risk perception.

Nearly half of the students reported participating in environmental activities. These activities were not observed to have any effect on their environmental attitudes; however, these students had higher risk perceptions. It may be possible that information and observations in these environmental activities may increase the risk perception of students. Moreover, in our study, students interested in environmental issues had more positive attitudes and higher level of risk perception. It is possible that students prefer to participate in activities that would have a positive impact on their attitude towards their interests.

In our study, the most common sources of information about environment were the internet, TV, and newspapers. The printed and visual media should fulfill their important role and accurately inform the public on environmental issues. However, sometimes media may act as a source of disinformation and exaggeration leading to misperceptions on the environment, which could be prevented by establishing an open communication line between the scientists and media programmers.

There was a positive correlation between the mean scores of EAS and ERPS among the students. Elevated EAS scores were associated with increased ERPS scores including the ecological, chemical waste, resource depletion, and global environmental risk factors. This finding suggests that any positive contribution in terms of developing a positive environmental attitude may affect risk perception.

In conclusion, we found positive environmental attitude and above moderate-level environmental risk perception in the Mersin University students. This positive attitude and above-moderate-level risk perception predominantly involved environmental issues threatening human health and welfare. The students expressed a lower level of interest towards ecological problems and issues that would have a negative effect on future generations. To improve the negative environmental attitudes of students, the university curriculum should be revised. Multidisciplinary studies should be performed to develop an environmental education program for students attending administrative sciences. Our study shows that environmental education is a life-long process that cannot be limited to educational institutions. NGOs, local administrations, and mass media have all significant functions in providing information and raising environmental awareness and consciousness on environment. All the state and private organizations should carry out coordinated activities to develop positive environmental attitude and raise the environmental risk perception.

## Figures and Tables

**Table 1:** The distribution of students according to sociodemographic characteristics and training and interest in environmental issues.

	*n*	%
*Sociodemographic characteristics*		
Sex (*n* = 774)		
Female	432	55.8
Male	342	44.2
Preuniversity place of residence (*n* = 769)		
Metropolitan	380	49.4
City	165	21.5
District	172	22.4
Rural	52	6.7
Residence (*n* = 766)		
Living with friends	298	38.9
Living with parents	222	29.0
Student hostel	161	21.0
Living alone	85	11.1
Mother's education (*n* = 750)		
Primary school and less	384	51.2
Secondary school	98	13.1
High school	184	24.5
University degree	84	11.2
Father's education (*n* = 753)		
Primary school and less	231	30.7
Secondary school	107	14.2
High school	235	31.2
University degree	180	23.9
Mother's occupation (*n* = 742)		
Working	142	19.1
Not working	600	80.9
Father's occupation (*n* = 732)		
Worker	155	21.2
Officer	111	15.2
Retired	168	23.0
Trade/self-employed	203	27.6
Professional	95	13.0
*Training and interest in environmental issues*		
Faculties (*n* = 774)		
Social Sciences	225	29.0
Health Sciences	201	26.0
Engineering and Technology	188	24.3
Educational Sciences	160	20.7
Received environmental education before university (*n* = 771)		
Yes	343	44.5
No	428	55.5
Received environmental education at the university (*n* = 770)		
Yes	408	53.0
No	362	47.0
Received an extracurricular activity (*n* = 770)		
Yes	191	24.8
No	579	75.2
Interested in environmental subjects (*n* = 771)		
Interested	623	80.8
Not interested	148	19.2
Member of an environmental organization (*n* = 771)		
Yes	111	14.4
No	660	85.6
Participating in environmental activities (*n* = 771)		
Yes	327	42.4
No	444	57.6

**Table 2:** Environmental Risk Perception Scale mean scores of students.

Risk items	Score Mean ± SD
(1)* Acid rain *caused by the deposition of acid-producing sulfur dioxide into streams and on forests, usually from the burning of coal	5.7 ± 1.5
(2)* Global warming *caused by excessive amounts of greenhouse gases like carbon dioxide and methane that may lead to weather extremes, such as temperature increases and flooding	5.7 ± 1.5
(3) *The ozone hole *caused by ozone-depleting substances like refrigerants (e.g., freon) that reduce the protective ozone layer and lead to an increase in ultraviolet radiation from the sun	5.7 ± 1.4
(4)* Drilling for oil *from offshore drilling platforms along the coasts and the transportation of oil and petroleum products (e.g., pipelines, tank trucks, and supertankers) that may result in spills	5.3 ± 1.6
(5)* Hazardous waste sites *which may release toxic chemicals into streams and estuaries and landscapes	5.9 ± 1.5
(6)* Radiation*: release of radioactive materials associated with nuclear power generation	6.0 ± 1.4
(7)* Persistent and toxic organic pollutants *(e.g., PCBs, DDT, dioxin, toluene, and benzene) that are discharged into surface streams or into the air from chemical manufacturing plants	6.0 ± 1.4
(8)* Heavy metals *like lead, zinc, and cadmium released into surface waters from mining operations and mercury released from the burning of coal	5.7 ± 1.4
(9)* Pesticides*: insecticides used to treat insect pests, herbicides used to treat weeds, and rodenticides used to kill animal pests	5.3 ± 1.5
(10)* Eutrophication*: the overenrichment of waters due to nitrogen fertilizer runoff and nitrogen oxide deposition in watersheds. This may lead to algal blooms and depletion of dissolved oxygen in rivers and coastal waters	5.4 ± 1.5
(11)* Sewage*: untreated sewage dumped from cruise ships and treated sewage from waste water treatment plants discharged into streams	5.8 ± 1.4
(12) The growing of *genetically engineered crops *(e.g., corn), also known as genetically modified organisms or GMOs	5.6 ± 1.5
(13) *Invasive species*: species that are not native to a specific location and have a tendency to spread, causing damage to the local environmental components	5.4 ± 1.5
(14) *Clear-cut logging *of large tracts of forests for pulp, paper, and wood products	5.4 ± 1.6
(15) *Destruction and fragmentation of wildlife habitat* due to urbanization and suburban sprawl	5.7 ± 1.4
(16) The *damming of rivers *for electric power generation, flood control, navigation, and recreation	5.1 ± 1.7
(17) Destruction and *loss of wetlands *by residential, commercial, industrial, agricultural, or recreational development	5.6 ± 1.5
(18)* Surface runoff *(also known as nonpoint pollution) contaminated with agricultural chemicals and sediment	5.5 ± 1.5
(19) *Overgrazing of range *and pasture lands due to excessive numbers of livestock for a specific area	4.7 ± 1.8
(20) *Sport and recreational hunting*	5.0 ± 1.8
(21) *Commercial fishing*	4.6 ± 1.8
(22) Worldwide human *population growth*	5.5 ± 1.5

**Table 3:** The highest and lowest mean scores of five situations based on risk perception to faculty.

	Risk perception: highest five situations		Risk perception: lowest five situations	
	Risk	Score	Risk	Score
		Mean ± SD		Mean ± SD
Health Sciences	Radiation	6.3 ± 1.2	Overgrazing	4.6 ± 1.9
	Organic pollutants	6.2 ± 1.2	Commercial fishing	4.7 ± 1.9
	Hazardous waste	6.2 ± 1.2	Damming of rivers	5.1 ± 1.8
	Sewage	6.0 ± 1.3	Hunting	5.2 ± 1.8
	Heavy metals	6.0 ± 1.3	Clear-cut logging	5.4 ± 1.6

Engineering and Technology	Radiation	6.1 ± 1.3	Overgrazing	4.6 ± 1.8
	Organic pollutants	6.1 ± 1.3	Commercial fishing	4.7 ± 1.7
	Hazardous waste	6.1 ± 1.3	Hunting	5.0 ± 1.8
	Sewage	6.0 ± 1.3	Damming of rivers	5.2 ± 1.5
	The ozone hole	5.9 ± 1.3	Pesticides	5.4 ± 1.3

Educational Sciences	Organic pollutants	5.9 ± 1.4	Commercial fishing	4.7 ± 1.6
	Radiation	5.9 ± 1.4	Overgrazing	5.0 ± 1.6
	Global warming	5.8 ± 1.3	Damming of rivers	5.1 ± 1.6
	Loss of wildlife habitat	5.8 ± 1.3	Hunting	5.2 ± 1.7
	Acid rain	5.8 ± 1.3	Pesticides	5.2 ± 1.4

Social Sciences	Radiation	5.6 ± 1.7	Commercial fishing	4.4 ± 1.8
	Organic pollutants	5.6 ± 1.6	Overgrazing	4.5 ± 1.9
	Sewage	5.5 ± 1.6	Hunting	4.8 ± 1.9
	GMOs	5.5 ± 1.7	Pesticides	5.0 ± 1.6
	Loss of wildlife habitat	5.5 ± 1.6	Damming of rivers	5.0 ± 1.8

Total	Radiation	6.0 ± 1.4	Commercial fishing	4.6 ± 1.8
	Organic pollutants	6.0 ± 1.4	Overgrazing	4.7 ± 1.8
	Hazardous waste	5.9 ± 1.5	Hunting	5.0 ± 1.8
	Sewage	5.8 ± 1.4	Damming of rivers	5.1 ± 1.7
	Heavy metals	5.7 ± 1.4	Pesticides	5.3 ± 1.5

**Table 4:** The distribution of Environmental Risk Perception Scale subscale scores according to the faculty.

	Ecological risks	Chemical waste risk	Resource depletion	Global environmental risks
	Mean ± SD	Mean ± SD	Mean ± SD	Mean ± SD
Health Sciences	5.7 ± 1.1^a^	6.0 ± 1.0^b,c^	5.0 ± 1.4	5.8 ± 1.2^e^
Engineering and Technology	5.5 ± 1.0	5.9 ± 1.0^d^	4.9 ± 1.3	5.7 ± 1.1^f^
Educational Sciences	5.4 ± 1.1	5.7 ± 1.1^c^	5.1 ± 1.2	5.7 ± 1.1^g^
Social Sciences	5.2 ± 1.3^a^	5.4 ± 1.3^b,d^	4.8 ± 1.4	5.3 ± 1.4^e,f,g^

Total	5.5 ± 1.1	5.8 ± 1.2	4.9 ± 1.3	5.6 ± 1.2

^a^ *p* = 0.002, ^b^*p* < 0.0001, ^c^*p* = 0.036, ^d^*p* < 0.0001, ^e^*p* < 0.0001, ^f^*p* = 0.002, and ^g^*p* = 0.014.

**Table 5:** Environmental Risk Perception Scale subscale scores from students related to characteristics.

	Ecological risks	Chemical waste risk	Resource depletion	Global environmental risks
	Mean ± SD	Mean ± SD	Mean ± SD	Mean ± SD
*Sex*				
Female	5.6 ± 1.1	5.9 ± 1.1	5.2 ± 1.3	5.8 ± 1.2
Male	5.2 ± 1.2	5.5 ± 1.2	4.6 ± 1.3	5.4 ± 1.3
	*p* < 0.001	*p* < 0.001	*p* < 0.001	*p* < 0.001
*Mother's occupation*				
Working	5.7 ± 1.2	6.0 ± 1.1	5.1 ± 1.4	5.8 ± 1.2
Not working	5.4 ± 1.1	5.7 ± 1.1	4.9 ± 1.3	5.6 ± 1.2
	*p* = 0.04	*p* = 0.03	*p* = 0.12	*p* = 0.05
*Interested in environmental subjects*				
Interested	5.6 ± 1.1	5.9 ± 1.1	5.1 ± 1.3	5.80 ± 1.22
Not interested	5.0 ± 1.3	5.3 ± 1.3	4.5 ± 1.4	5.03 ± 1.41
	*p* < 0.001	*p* < 0.001	*p* < 0.001	*p* < 0.001
*Participating in environmental activities*				
Yes	5.6 ± 1.1	5.9 ± 1.1	5.1 ± 1.3	5.7 ± 1.2
No	5.4 ± 1.2	5.7 ± 1.2	4.8 ± 1.3	5.5 ± 1.3
	*p* = 0.01	*p* = 0.03	*p* = 0.01	*p* = 0.02
